# Commensurate antiferromagnetic excitations as a signature of the pseudogap in the tetragonal high-*T*_c_ cuprate HgBa_2_CuO_4+δ_

**DOI:** 10.1038/ncomms10819

**Published:** 2016-03-04

**Authors:** M. K. Chan, C. J. Dorow, L. Mangin-Thro, Y. Tang, Y. Ge, M. J. Veit, G. Yu, X. Zhao, A. D. Christianson, J. T. Park, Y. Sidis, P. Steffens, D. L. Abernathy, P. Bourges, M. Greven

**Affiliations:** 1 School of Physics and Astronomy, University of Minnesota, Minneapolis, Minnesota 55455, USA; 2 Pulsed Field Facility, National High Magnetic Field Laboratory, Los Alamos National Laboratory, Los Alamos, New Mexico 87545, USA; 3 Laboratoire Léon Brillouin, LLB/IRAMIS, UMR12, CEA-CNRS, CEA-Saclay, Gif sur Yvette 91191, France; 4 State Key Lab of Inorganic Synthesis and Preparative Chemistry, College of Chemistry, Jilin University, Changchun 130012, China; 5 Quantum Condensed Matter Division, Oak Ridge National Laboratory, Oak Ridge, Tennessee 37831, USA; 6 Forschungsneutronenquelle Heinz Maier-Leibnitz, Garching 85747, Germany; 7 Institute Laue Langevin, Grenoble 38042 CEDEX 9, France

## Abstract

Antiferromagnetic correlations have been argued to be the cause of the *d*-wave superconductivity and the pseudogap phenomena exhibited by the cuprates. Although the antiferromagnetic response in the pseudogap state has been reported for a number of compounds, there exists no information for structurally simple HgBa_2_CuO_4+δ_. Here we report neutron-scattering results for HgBa_2_CuO_4+δ_ (superconducting transition temperature *T*_c_≈71 K, pseudogap temperature T*≈305 K) that demonstrate the absence of the two most prominent features of the magnetic excitation spectrum of the cuprates: the X-shaped ‘hourglass' response and the resonance mode in the superconducting state. Instead, the response is Y-shaped, gapped and significantly enhanced below T*, and hence a prominent signature of the pseudogap state.

## Abstract (web summary)

In the cuprates, antiferromagnetic correlations might be the cause of the pseudogap phenomenon. Here the authors use neutron scattering on the tetragonal cuprate HgBa_2_CuO_4+δ_ revealing commensurate antiferromagnetic excitations as a signature of the pseudogap state.

## Graphical abstract

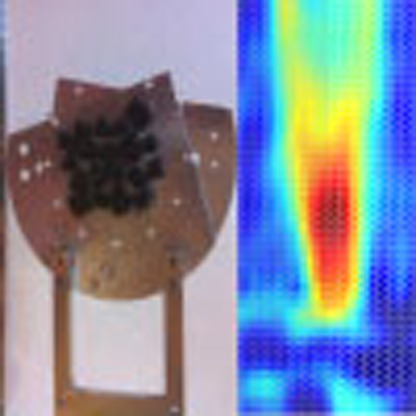

Most of the detailed knowledge about magnetic fluctuations in the cuprates comes from neutron-scattering studies of La_2-*x*_Sr_*x*_CuO_4_ (LSCO) and YBa_2_Cu_3_O_6+*y*_ (YBCO)^1^,^2^. The momentum (**Q)** and energy- (*ω*) dependent dynamic magnetic susceptibility *χ″*(**Q**,*ω*) of LSCO is characterized by an X-shaped hourglass spectrum that disperses with increasing energy from incommensurate wave vectors at *ω*≈0 towards the antiferromagnetic wave vector **q**_AF_, and then outward again at higher energies^1^,^3^. YBCO exhibits a similar dispersion in the superconducting (SC) state, yet its foremost characteristic is the magnetic resonance, a large and abrupt susceptibility increase at **q**_AF_ and a well-defined energy *ω*_**r**_ upon cooling below *T*_c_ (ref. 2). Despite the apparent ubiquity of the hourglass dispersion, the differences between the spectra for LSCO and YBCO have motivated ostensibly contradictory microscopic interpretations: incommensurate spin-density-wave (SDW) fluctuations of local moments (unidirectional charge-spin stripe order is one example)^4^, or a spin-exciton because of particle–hole excitations in the SC state^2^,^5^. The persistence of low-energy incommensurate excitations at temperatures well above *T*_c_ for both LSCO and YBCO has stimulated speculation that the opening of the pseudogap (PG) is associated with SC^6^ or SDW^1^,^7^,^8^ fluctuations.

Although it has long been known that AF correlations may cause the *d*-wave superconductivity exhibited by the cuprates^9^, such theoretical approaches were not thought to be able to account for the PG phenomenology. Following recent theoretical advancements^10^, it has been argued that AF correlations may not only drive *d*-wave superconductivity, but potentially also PG electronic instabilities such as charge order (charge-density-wave (CDW), bond-density-wave), translational symmetry-preserving (**q**=0) loop-current order, and pair-density-wave order^11^,^12^,^13^,^14^. These developments raise the prospect that much of the cuprate phase diagram may be understood as driven by AF correlations. It is therefore imperative to determine the detailed magnetic response in a structurally simple compound in which both CDW^15^ and **q**=0 (refs 16, 17) order have been firmly established. Such measurements might also help illuminate the relevance of the seemingly universal hourglass response.

HgBa_2_CuO_4+δ_ (Hg1201) features the highest optimal *T*_c_ (*T*_c,max_=97 K) of the single-CuO_2_-layer cuprates (for example, *T*_c,max_=39 K for LSCO; YBCO has *T*_c,max_=93 K and a double-CuO_2_-layer structure), a simple tetragonal crystal structure, and minimal disorder effects^18^. Similar to the recent demonstration of the validity of Kohler's rule for the magnetoresistance in the PG phase^19^, these model-system characteristics of Hg1201 can be expected to most clearly reveal the inherent magnetic fluctuation spectrum of the quintessential CuO_2_ layers.

We present an inelastic neutron-scattering study of the magnetic excitations of an underdoped Hg1201 sample with *T*_c_≈71 K and hole doping *p*≈0.095 (labelled HgUD71; see Methods, Supplementary Notes 1–2 and Supplementary Figs 1–5 for detailed experimental and analysis information). This is a particularly interesting doping level because it corresponds to the shoulder of the ‘SC dome', where *T*_c_ appears to be slightly suppressed, and because a cascade of phenomena have been observed: quasi-static **q**=0 magnetic order^16^,^17^ below T*, short-range CDW correlations^15^ below *T*_CDW_≈200 K, evidence for Fermi-liquid transport in the PG state^19^,^20^,^21^, and Shubnikov-de Haas oscillations (below 4 K in magnetic fields above 60 T) (ref. 22). The slight suppression of *T*_c_ at this doping level might be a signature of a competing ground state, and it is interesting to determine if this has an effect on the dynamic magnetic susceptibility. As shown in Fig. 1a, the situation for Hg1201 mirrors the phenomenology of underdoped YBCO^23^,^24^,^25^,^26^,^27^,^28^. We find that the dynamic magnetic response of HgUD71 is characterized by a gapped Y-shaped dispersion that is commensurate with **q**_AF_ at energies below about 60 meV. Interestingly, the magnetic scattering exhibits a marked increase below the PG temperature T* yet is largely impervious to the onset of CDW order and of superconductivity. This establishes the commensurate excitations as a signature of the PG state.

## Results

### Time-of-flight neutron spectroscopy

Figures 2a,b shows *χ*″(**Q**,*ω*) at 5 and 85 K for HgUD71 extracted from raw neutron-scattering data. At 5 K, deep in the SC phase, the susceptibility exhibits a prominent peak at *ω*_peak_≈51 meV and is gapped below Δ_AF_≈27 meV (see Supplementary Notes 3 and Supplementary Fig. 6 for limits on the low-energy scattering). Up to approximately *ω*_com_=59 meV, the response is commensurate with **q**_AF_, and then it disperses outward at higher energies, resulting in a gapped Y-shaped spectrum (Figs 2a,c–g). The magnetic nature of the response is confirmed through spin-polarized neutron scattering and also from its **Q** dependence, which follows the magnetic form factor (see Supplementary Notes 4 and Supplementary Fig. 7).

We fit constant-**Q** data such as those in Fig. 2c–l to a Gaussian form, 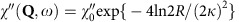, convolved with the momentum resolution of the instrument (Supplementary Fig. 2), where *R*=|[(*H*-1/2)^2^+(*K*-1/2)^2^]^1/2^-*δ*|^2^, 2κ is the full-width at half-maximum (FWHM), and *δ* parameterizes the incommensurability away from **q**_AF_. The energy dependences of 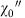, 2κ and *δ* are shown in Fig. 3a,b inset, and Fig. 3e, respectively.

### Commensurate low-energy magnetic excitations

As shown in Fig. 2, the low-energy magnetic excitations in HgUD71 are commensurate with **q**_AF_. In Fig. 3e, δ=0 for *ω*<*ω*_com_, since this results in the best fit to the data. We also fit the data with *δ*≠0 (Fig. 4a) to facilitate comparison with published results for YBCO and LSCO (Fig. 4b,c), where at the neck of the hourglass a non-zero value of δ is typically employed in the data analysis even when the response is essentially commensurate. We find an upper bound of *δ*≈0.03 r.l.u. for HgUD71 (*ω*<*ω*_com_), which is consistent with the half-width at half-maximum of the instrumental momentum resolution (Fig. 4a and Supplementary Fig. 2). As seen from Fig. 4b,c, this upper bound is significantly smaller than the incommensurability observed in both LSCO and YBCO at similar doping levels.

### Evolution of magnetic excitations across T* and *T*_c_

Figure 3a shows the energy dependence of the susceptibility amplitude 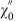 at four temperatures. A comparison of the data at 5 and 85 K reveals hardly any effect of superconductivity. This is also apparent from Fig. 3c, which shows the change 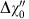 between these two temperatures. As seen from Fig. 3a, a further increase of temperature suppresses 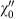 at all measured energies (up to 53 meV for *T>*85 K).

**Figure 1:**
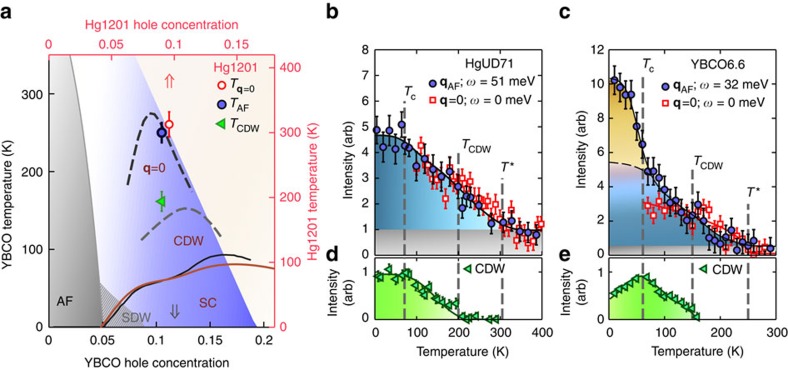
Phase diagram of Hg1201 and YBCO. (**a**) Phase diagram: Hg1201 (red top-right axes) and YBCO (black bottom-left axes). The axes are adjusted such that the *T*_c_(*p*) domes of Hg1201 (red line) and YBCO (black line) approximately line up. The blue region represents the PG regime. The red and grey arrows indicate the doping of HgUD71 (*T*_c_=71 K) and YBCO6.6 (*T*_c_=61 K) highlighted in this work. Dashed black and grey lines represent the temperatures below which **q**=0 magnetic order^23^,^24^ and CDW^25^,^26^,^27^ correlations are observed in YBCO. The corresponding data for Hg1201 (*T*_**q**=0_, red circle; *T*_CDW_, green triangle) for doping levels close to HgUD71 are shown^15^,^16^,^17^. The significant increase in the AF response appears below *T*_AF_ (blue circle). Grey shaded and hashed areas represent AF and SDW^28^ order in YBCO. (**b**) Temperature dependence of inelastic magnetic scattering at **q**_AF_ and *ω*_peak_=51 meV and of quasi-elastic (FWHM energy resolution ∼1 meV) magnetic scattering at **q**=0 (from ref. 17) measured on separate Hg1201 samples with similar doping levels (*T*_c_=71 and 75 K, respectively). We determine *T*_**q**=0_=320±20 K (ref. 17) and *T*_AF_=300±15 K. The **q**=0 signal is shifted upward (by one unit) for comparison. Both *T*_**q**=0_ and *T*_AF_ are consistent with T*= 305±10 K determined from the planar resistivity (deviation from high-temperature linear dependence)^19^. (**c**) Temperature dependence of the **q**=0 signal^23^ and of the odd-parity resonance (*ω*_r_=32 meV) for a twinned YBCO6.6 sample (see Methods for sample details). For **b**,**c**, the blue region represents the increase in intensity in the PG state and the grey region is the baseline intensity for *T* > T*. The shaded orange region in **c** represents the excess scattering below *T*_c_ because of the resonance mode. (**d**,**e**) Temperature dependence of short-range CDW order in Hg1201 (ref. 15) and YBCO (ref. 26) at approximately the same respective doping levels as the data in **b**,**c**. Vertical error bars in **a**–**c** are statistical errors (1 s.d.).

To better ascertain the temperature dependence of magnetic excitations, we focus on the response at *ω*_peak_≈51 meV and **q**_AF_ (Fig. 1b). Consistent with Fig. 3a, the intensity does not exhibit an abrupt change across *T*_c_, which confirms the lack of a magnetic resonance. However, a marked increase in intensity occurs below the PG temperature T*. For comparison, we measured the temperature dependence of the intensity of the odd-parity resonance mode of YBCO at a similar doping level (sample labelled YBCO6.6: *y*=0.6, *T*_c_=61 K, *p*=0.11 and *ω*_r_=32 meV; see black arrow in Fig. 1a). The result is shown in Fig. 1c. As for HgUD71, the intensity for YBCO6.6 increases below T*, yet in contrast to HgUD71, a large magnetic resonance is observed below *T*_c_.

**Figure 2:**
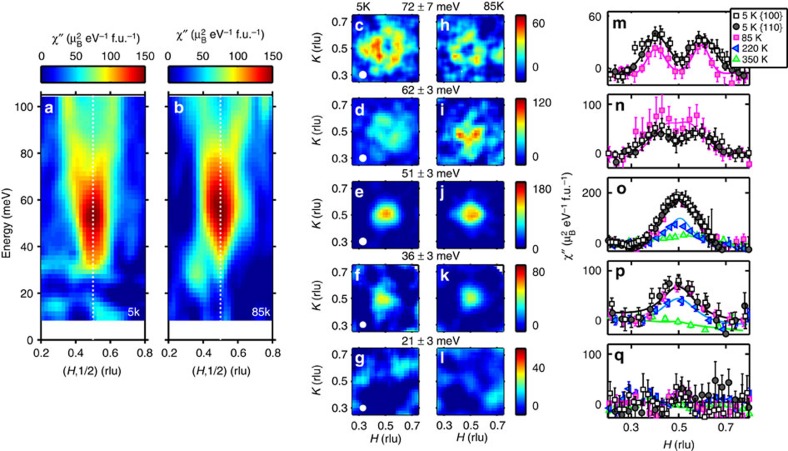
Magnetic excitation spectrum of HgUD71 features gapped, commensurate and dispersive components. (**a**,**b**) Energy dependence of *χ*″(**Q**,*ω*) for HgUD71 at 5 and 85 K (14 K above *T*_c_), respectively, along the two-dimensional momentum-transfer trajectory [*H*,0.5], with intensity averaged over the range *K*=0.5±0.12. The gap Δ_AF_ is defined as the energy below which no scattering is observed at **q**_AF_; Δ_AF_≈27 meV at both 5 and 85 K. (**c**–**l**) Constant-energy slices of magnetic scattering at *T*=5 K in **c**–**g** and *T*=85 K in **h**–**l**. (**m**–**q**) The corresponding constant-energy cuts along high-symmetry trajectories. Cuts along {100} (average of [H00] and [0H0] cuts) and {110} (average of [HH0] and [H-H0] cuts) are shown for *T*=5 K (open black squares and closed black circles, respectively). Data at higher temperatures (85, 220 and 350 K) are averages of four cuts along [H00], [0H0], [HH0] and [H-H0] trajectories. Error bars represent statistical error (1 s.d.). The white circles in **c**–**g** represent the momentum resolution at the corresponding energy transfers. Data collected on ARCS (see Methods).

**Figure 3:**
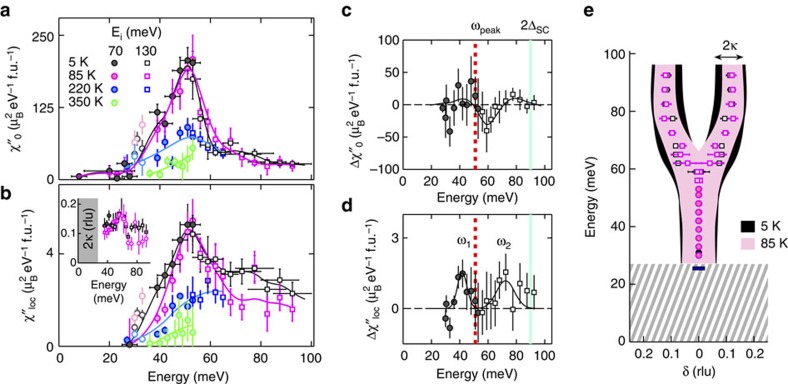
Magnetic susceptibility amplitude and local susceptibility for HgUD71. (**a**) Energy dependence of the measured peak magnetic susceptibility 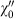 at *T*=300, 220, 85 and 5 K. Closed circles: *E*_i_=70 meV. Open squares: *E*_i_=130 meV. Solid lines: guides to the eye. Horizontal bars for the 5 K data represent energy bins. The same binning is used at higher temperatures. Between 30 and 33 meV the data are systematically contaminated by aluminium and phonon scattering, and are represented as lighter open symbols (see Supplementary Fig. 3). (**b**) Same legend as **a**. Energy dependence of the momentum-integrated (local) susceptibility *χ*″_loc_. In determining *χ*″_loc_, we assume that AF fluctuations are quasi-two-dimensional, that is, that χ″ does not depend on *L*. Inset: 2*κ* (FWHM) as a function of energy at 5 K (black) and 85 K (magenta). (**c**,**d**) Change of 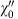 and *χ*″_loc_, respectively, between 5 and 85 K (that is, across *T*_c_). Filled and open symbols: *E*_i_=70 and 130 meV, respectively. The red vertical line marks *ω*_peak_. The turquoise line represents 2Δ_SC_, where Δ_SC_=45±1 meV is the maximum SC *d*-wave gap determined from Raman scattering^33^. Black line in **c**: guide to the eye. Black line in **d**: fit to two Gaussian peaks, located at *ω*_1_=44±2 meV and *ω*_2_=75±2 meV. (**e**) Energy dependence of incommensurability δ at 5 K (black) and 85 K (red). Horizontal error bars are fit uncertainties for *δ*. For *ω*<59 meV, the data are best described with *δ*=0. We estimate an upper bound of *δ*≈0.03, which is the approximate value of the instrumental momentum resolution in the *ω*=27–59 meV range. Shaded black and magenta regions represent 2*κ* at 5 and 85 K, respectively. Hatched area indicates the gap Δ_AF_. Horizontal blue bar at *ω*=27 meV represents the instrumental momentum resolution at that energy for *E*_i_=70 meV. All vertical error bars in figure are least-square fit errors (1 s.d.).

Even though HgUD71 does not exhibit a magnetic resonance, upon cooling into the SC phase we observe subtle changes in the susceptibility at wave vectors away from **q**_AF_ and at energies below and above *ω*_peak_, centred at *ω*_1_=44 meV and *ω*_2_=75 meV. This is best seen from the momentum-integrated local susceptibility, 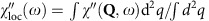 (Fig. 3b,d). At *ω*_1_, the change across *T*_c_ (Δ*χ*″_loc_) is due to a slight increase of both the momentum width and intensity, whereas at *ω*_2_ it results from an increase in amplitude on the upward dispersive part of the spectrum (see also Fig. 2c,h,m). The **Q** dependence of these subtle changes across *T*_c_ is discussed in more detail in Supplementary Note 5 and Supplementary Fig. 8.

## Discussion

**Figure 4:**
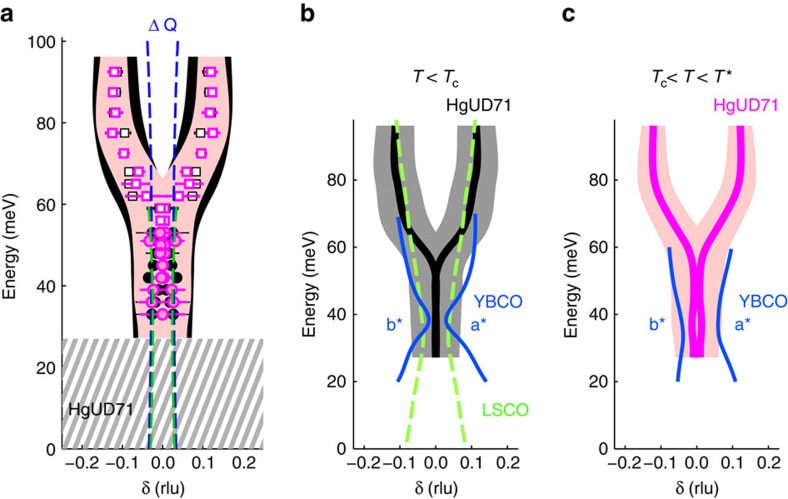
Comparison of Y-shaped response of HgUD71 with magnetic excitation spectrum of YBCO and LSCO at similar doping levels. (**a**) Energy dependence of incommensurability *δ* at 5 K (black) and 85 K (magenta), with *δ* assumed to be non-zero at all energies. We arrive at an upper bound of *δ*∼0.03 r.l.u., which corresponds to the half-width at half-maximum of the instrumental **Q** resolution (FWHM resolution for *E*_i_=70 and 130 meV indicated by the green and blue dotted lines, respectively). Shaded black and red regions represent the measured FWHM (2*κ*) at 5 and 85 K, respectively. Hatched grey area indicates the gap in the excitation spectrum. (**b**) Comparison of the dispersion of HgUD71 with YBCO6.6 (ref. 7, 8) and LSCO (*p*=*x*=0.085) (ref. 3) deep in the SC state. (**c**) Comparison of the dispersion of HgUD71 with YBCO6.6 above *T*_c_ (*T*=85 and 70 K, respectively)^7^. The response of orthorhombic YBCO6.6 (blue lines) is anisotropic, and therefore *δ* along both a* (right) and b* (left) is shown. As in **a**, the shaded regions in **b**,**c** indicate the momentum widths (FWHM) of the response of HgUD71.

The absence of an hourglass dispersion in HgUD71 constitutes a clear departure from the purported universal magnetic response of the cuprates. Figure 4 compares the **Q**-*ω* dispersion of the magnetic fluctuations centred at **q**_AF_ at similar doping levels for HgUD71, YBCO6.6 (refs 7, 8) and LSCO (*p*≈0.085, *T*_c_=22 K)^3^; see Supplementary Fig. 9 for an additional comparison between HgUD71 and LSCO (*p*≈0.085). LSCO exhibits a gapless hourglass dispersion both in the SC state and in the normal state. In the local moment picture, the incommensurate low-energy response is argued to be a signature of SDW correlations^1^. For underdoped LSCO^1^ and YBCO^28^, the occurrence of incommensurate SDW order revealed by neutron-scattering correlates with a planar resistivity characterized by a sizable extrapolated zero-temperature residual and by a low-temperature insulating-like upturn (when superconductivity is suppressed with large magnetic fields) below a non-universal critical doping *p*_c_; *p*_c_≈0.16 for LSCO^29^ and *p*_c_≈0.085 YBCO^30^. The doping-temperature range of the SDW correlations in YBCO is shown in Fig. 1a. At the doping level of our study, Hg1201 exhibits electrical transport without a significant zero-temperature residual^20^, Kohler scaling of the normal state magnetoresistance^19^, as well as quantum oscillations^22^, which demonstrates an underlying metallic ground state. Although *p*_c_ for Hg1201 is not known, it is likely smaller than *p*≈0.055 (*T*_c_=45 K), for which the residual resistivity is still very small^20^. In addition to the commensurate low-energy response reported here for HgUD71, this indicates that Hg1201 is less prone to SDW order than YBCO and especially LSCO.

In the SC state, YBCO^7^ (for *p>p*_c_) exhibits a prominent resonance and a gapped magnetic response that is hourglass-shaped (Figs 1c, 4b). The hourglass dispersion and resonance are best explained as signatures of the *d*-wave SC order parameter within the itinerant spin-exciton picture^2^,^5^. Although the resonance is well established over a wide doping range in double-layer YBCO^2^, for single-layer compounds (Tl_2_Ba_2_CuO_6+δ_ (ref. 2) and Hg1201 (ref. 31)) it has been reported only close to optimal doping, where the PG phenomenon is less prominent. According to the relationship *ω*_r_/2Δ_SC_=0.64±0.04 found for unconventional superconductors^32^, and with the estimate 2Δ_SC_=78–91 meV from electronic Raman scattering^33^ and photoemission spectroscopy^34^, we expect *ω*_r_≈54 meV, which is close to *ω*_peak_≈51 meV for HgUD71. Interpreted within the spin-exciton picture, the suppression of the magnetic resonance at **q**_AF_ might result from an absence of coherent Bogoliubov quasi-particles at the ‘hotspots' (where the underlying Fermi surface intersects the AF Brillouin zone boundary) as a result of the antinodal PG. This is consistent with electronic Raman scattering for Hg1201 (refs 33, 35), namely the fact that the SC pair-breaking peak in the *B*_1g_ channel, which probes the antinodal states, significantly weakens upon underdoping (samples with *T*_c_ below about 78 K), whereas the peak in the *B*_2g_ channel, which probes the nodal states, persists. It is furthermore consistent with our observation of an increase of *χ*″(**Q**,*ω*) below *T*_c_ at momenta away from **q**_AF_ that connect parts of the Fermi-surface closer to the coherent nodal directions that are unaffected by the PG.

The significant increase of the magnetic response below T* (Fig. 1b) and the concomitant absence of a prominent effect across *T*_c_ indicates that the AF response for HgUD71 is dominated by the PG formation. The latter is a pivotal characteristic of the cuprates, and it is possibly associated with an underlying quantum critical point that controls much of the phase diagram^36^,^37^. A close connection between *χ*″(**Q**,*ω*) and the PG has been suggested before^3^,^6^,^7^. In early work on YBCO, it was argued that the magnetic fluctuations in the PG state are a precursor of the resonance and therefore a signature of fluctuating superconductivity^6^. However, more recent work^7^ on detwinned YBCO6.6 found that *χ*″(**Q**,*ω*) in the PG state is in fact distinct from that in the SC state. Furthermore, the broken fourfold structural symmetry of YBCO results in a large anisotropy in *χ*″(**Q**,*ω*) for the two in-equivalent planar crystallographic directions. Whereas the dispersion along [010] is reminiscent of the commensurate Y-shaped spectrum of HgUD71, the response along [100] is broader and incommensurate at low energies (Fig. 4c) (ref. 7). This led to speculation that the PG is characterized either by stripe fluctuations, similar to LSCO, or by a nematic instability^7^. However, an alternative explanation for the anisotropy is interlayer coupling to the unidirectional CuO chain states of YBCO^38^, a complication that is absent in HgUD71. Our result for this structurally simpler cuprate, showing a PG state characterized by a commensurate and isotropic low-energy magnetic response with no connection to a magnetic resonance, calls for a new theoretical interpretation. We speculate that the commensurate response for *ω*<*ω*_com_ predominantly results from particle–hole scattering near the AF hotspots, and that it involves the non-dispersive region in the spectral density of states determined from scanning tunnelling microscopy in the PG state^39^.

A number of broken symmetries have been identified in the PG regime. In particular, the cuprates exhibit strong (∼0.1 μ_B_) **q**=0 quasi-elastic magnetism^16^,^17^,^23^,^24^ that is qualitatively consistent with intra-unit-cell loop-current order^37^. We demonstrate in Fig. 1b that the significant enhancement of fluctuations at **q**_AF_ coincides with the onset of **q**=0 magnetism for HgUD71, which establishes a connection between these two seemingly distinct magnetic phenomena and with the opening of the PG at T*. On the other hand, (short-range) CDW correlations first appear at a temperature that is distinctly lower than T* (ref. 15) and have no discernible effect on the magnetic fluctuations (Fig. 1b,d, Supplementary Notes 6, Supplementary Fig. 10). Regarding the changes across T*, our result suggests that the development of AF correlations is a consequence rather than the cause of the PG. Nevertheless, these correlations might drive the subsequent CDW order, which in turn drives the Fermi-surface reconstruction implied by transport experiments in high magnetic fields^15^,^22^,^30^. It will be important to assess if this can indeed be the case given an instantaneous magnetic correlation length (estimated from integration over the measured energy range) of about two to three lattice constants in HgUD71.

Figure 1b–e show that, similar to HgUD71, for YBCO6.6 the intensity of the response at **q**_AF_ increases substantially along with the onset of **q**=0 order at T*. In contrast to HgUD71, for YBCO6.6 this is followed by a large resonance below *T*_c_ and by the concomitant appearance of the low-energy hourglass structure^7^,^8^ and of a significant suppression of the CDW response^26^. This indicates differing relative strengths of the SC and PG order parameters at temperatures below *T*_c_ for the two cuprates.

In summary, the AF response of the underdoped cuprates can be divided into three distinct types: (1) the gapless X-shaped spectrum associated with incommensurate SDW correlations of local moments in the La-based compounds^1^ and in lightly doped YBCO^28^, where **q**=0 magnetism is suppressed because of the competing SDW instability^24^; (2) the gapped X-shaped spectrum and magnetic resonance attributed to particle–hole excitations in the SC state^2^,^5^; and (3) the gapped Y-shaped spectrum associated with the PG formation (and with **q**=0 magnetism^16^,^17^ and metallic charge transport^19^,^20^,^22^) revealed most clearly in tetragonal Hg1201. The balance between PG, SDW and SC order parameters determines the magnetic response for a particular compound, doping level and temperature. We note that similar to LSCO, single-layer Bi_2+*x*_Sr_2-*x*_CuO_6+*y*_ (Bi2201) exhibits a propensity towards SDW order^40^, whereas double-layer Bi_2_Sr_2_CaCu_2_O_8+δ_ (Bi2212) near optimal doping features a dispersive resonance reminiscent of YBCO^41^. Just as for LSCO, *T*_c,max_=38 K (ref. 18) for Bi2201 is relatively low. Interestingly, the magnetic response of single-layer Hg1201 more closely resembles that of double-layer YBCO than those of single-layer LSCO and Bi2201. Yet the dominant PG behaviour is most clearly apparent in Hg1201, which does not feature the complications of YBCO because of the orthorhombic double-layer structure (even vs odd-parity magnetic excitations; in-equivalent response along [100] and [010]). To build a connection with the distinct magnetic response of the low-*T*_c,max_ single-layer compounds LSCO and Bi2201, it might be necessary to study Hg1201 with intentionally introduced disorder. Furthermore, experiments on Hg1201 at lower doping levels will be necessary to ascertain if the SDW instability is in fact a universal property of the cuprates.

## Methods

### Sample preparation

Single crystals of HgBa_2_CuO_4+δ_ were grown by a two-step self-flux method^42^. As-grown crystals are typically underdoped, with *T*_c_≈81 K. To reach the desired doping level, the crystals were annealed at 400 °C in a partial vacuum of 100 mtorr for 80 days^43^. The SC transition temperature of the individual crystals was subsequently determined from measurements of the Meissner effect in a SQUID magnetometer: each crystal was cooled in zero magnetic field, and the susceptibility was monitored upon warming in a 5 Oe field applied along the crystallographic *c* axis. Supplementary Fig. 1 shows the average susceptibility of all the 34 crystals that made up the HgUD71 sample. We find *T*_c_≈71 K (defined as the midpoint of the transition) with a full transition width of Δ*T*_c_=5 K for the assembled sample, and estimate *p*≈0.095 based on our thermoelectric power measurements of crystals from the same annealing batches. The doping level we estimate from these measurements is 0.005 higher than that estimated from prior published values for powder samples with the same *T*_c_ (ref. 44). The rather narrow combined transition width indicates a high degree of homogeneity and quality of the sample. We note that smaller crystals from the same growth and annealing batches exhibit Shubnikov-de Hass oscillations^22^. The fact that quantum oscillations can be observed at low temperatures is a consequence of the very small residual resistivity exhibited by these crystals^20^. The 34 crystals, with masses ranging from ∼20 to 125 mg, were polished parallel to the *ab*-plane and co-aligned on two aluminium plates with GE-varnish using a Laue backscattering X-ray machine. The resultant sample had a total mass of ∼1.6 g and a planar mosaic of about 2°. The plates were mounted on an aluminium sample holder, as shown in Supplementary Fig. 1b. Gadolinium oxide powder and cadmium plates (both Ga and Cd are strong neutron absorbers) were used to mask the excess aluminium.

The YBCO6.6 sample (data in Fig. 1c) was previously measured in refs 23, 45. The sample was grown with a top-seed melt texturing method and heat-treated to an underdoped state with *T*_c_=61±2.5 K. We estimate the doping level to be *p*=0.11 from the *T*_c_ versus doping relation in ref. 46.

### Definition of wave vector

We quote the scattering wave-vector **Q**=*H*a*+*K*b*+*L*c* as (*H*, *K*, *L*) in reciprocal lattice units (r.l.u.), where a*=b*=1.62 Å^−1^ and c*=0.66 Å^−1^ are the room-temperature magnitudes. The reduced two-dimensional wave vector is **q**=*h*a*+*k*b* and **q**_AF_=(1/2,1/2) r.l.u..

### Time-of-flight measurements

The time-of-flight measurements were performed with the ARCS spectrometer at the Spallation Neutron Source, Oak Ridge National Laboratory. The HgUD71 sample was mounted such that the incoming beam was parallel to the *c* axis of the sample. This means that for a particular in-plane wave vector (*H*, *K*), the out-of-plane component *L* depends on the energy transfer. Two measurement configurations were used: incident energies *E*_i_=70 and 130 meV, with Fermi-chopper frequencies of 420 and 600 Hz, respectively. The energy and momentum resolutions as a function of the energy transfer are presented in Supplementary Fig. 2. The out-of-plane wave-vector varies monotonically from *L*≈2–8 between *ω*=10–100 meV. As described in Supplementary Notes 1 and 2, the data are processed to isolate the AF fluctuations, and normalized by the magnetic form factor and Bose population factor to obtain *χ*″(**Q**,*ω*). Inherent to our analysis is the assumption that the magnetic response arises from the quintessential CuO_2_ planes and hence is quasi-two-dimensional, and that corrections for the *L* dependence can be made by accounting for the Cu magnetic form factor.

### Triple-axis measurements with unpolarized neutrons

Measurements on HgUD71 were performed with the HB3 spectrometer at the High-Flux Isotope Reactor at Oak Ridge National Laboratory (Fig. 1b). Measurements on YBCO6.6 (Fig. 1c) were performed with the 2T spectrometer at the Laboratoire Léon Brillouin (LLB, France) on the same twinned YBCO crystal (*T*_c_=61±2.5 K, *p*=0.11) used to measure the **q**=0 magnetic order in ref. 23. Pyrolytic graphite (PG) monochromators and analysers were used to select incident and final neutron energies, and PG filters were used to suppress contamination due to higher harmonics. The samples were mounted in the (*HHL*) scattering plane. Measurements were performed with fixed final energies *E*_f_=14.7 meV (HB3), and 35 meV (2T). On HB3, the horizontal collimation configuration was 48′-80′-sample-80′-120′. On 2T, no collimation was used, since vertical and horizontal focusing was employed at the monochromator. The typical energy resolution in the *ω*=50–60 meV energy transfer range was ∼8 meV.

## Additional information

**How to cite this article:** Chan, M. K. *et al*. Commensurate antiferromagnetic excitations as a signature of the pseudogap in the tetragonal high-*T*_c_ cuprate HgBa_2_CuO_4+δ_. *Nat. Commun.* 7:10819 doi: 10.1038/ncomms10819 (2016).

## Supplementary Material

Supplementary Information Supplementary Figures 1-10, Supplementary Notes 1-6 and Supplementary References.
