# Supplementary material for: Commensurate antiferromagnetic excitations as a signature of the pseudogap in the tetragonal high-Tc cuprate HgBa2CuO4+δ
Source: Nat Commun. 2016 Mar 4;7:10819. doi: 10.1038/ncomms10819 (PMC4785222; doi:10.1038/ncomms10819)
Supplement: Supplementary Information — Supplementary Figures 1-10, Supplementary Notes 1-6 and Supplementary References. [file ncomms10819-s1.pdf]

## Supplementary Information:

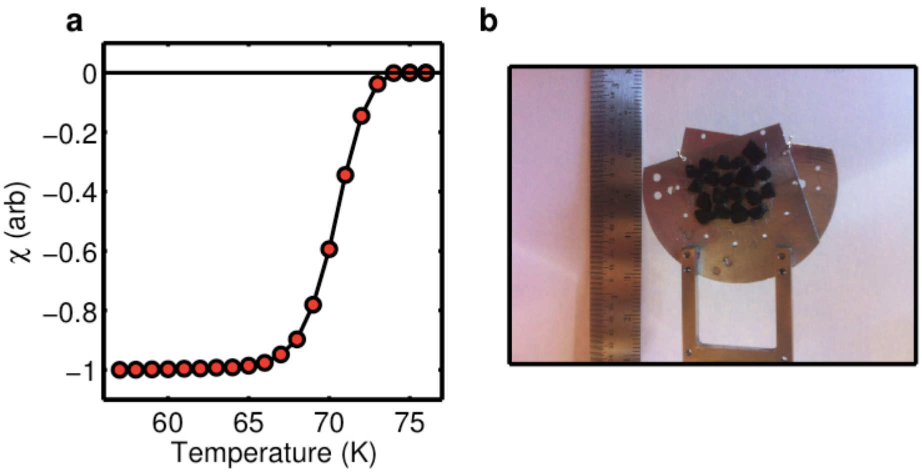


**Supplementary Figure 1 | Sample HgUD71.** (**a**) Average magnetic susceptibility of the constituent 34 crystals of sample HgUD71 with mid-point transition temperature $T_c$ = 71 K. (**b**) Picture of the two Aluminum plates with mounted crystals.

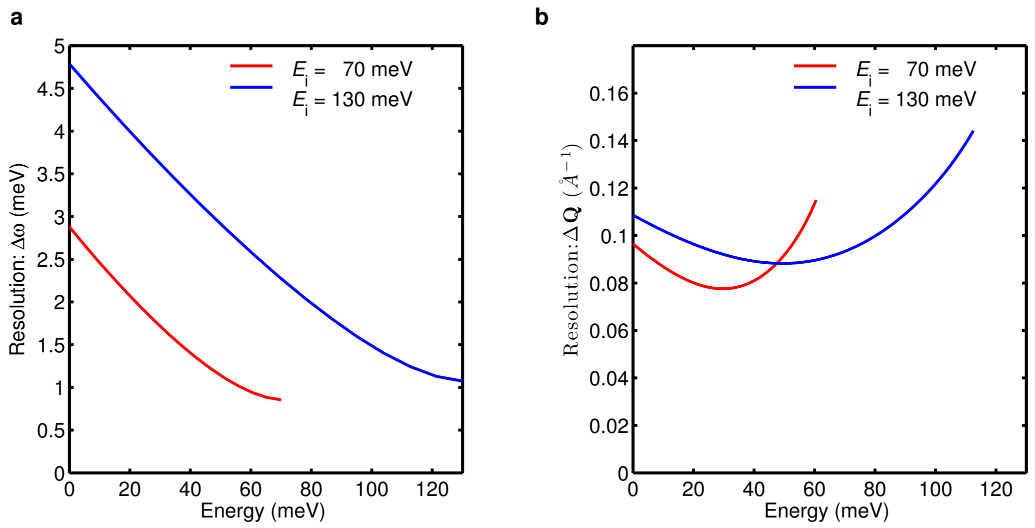


**Supplementary Figure 2 | ARCS instrumental resolution.** (**a**) Full-width-at-half-maximum (FWHM) energy resolution $\Delta\omega$ as a function of the energy transfer, determined by considering the contributions to the timing uncertainty from the source and chopper openings in conjunction with the neutron flight-path lengths[1]. The energy resolution improves as the energy transfer approaches the incident energy, $E_i$. (**b**) FWHM momentum resolution determined at the two-dimensional antiferromagnetic wave vector $\mathbf{q}_{AF}$ = (1/2, 1/2). Note that 1 r.l.u. = $2\pi/a \approx 1.63$ Å$^{-1}$, so that 0.08 Å$^{-1}$ ≈ 0.05 r.l.u. A sample mosaic of 2° was considered in the determination of the momentum resolution. The calculated energy and momentum resolutions were confirmed by comparing to the measured widths of the incoherent scattering and of elastic Bragg peaks.

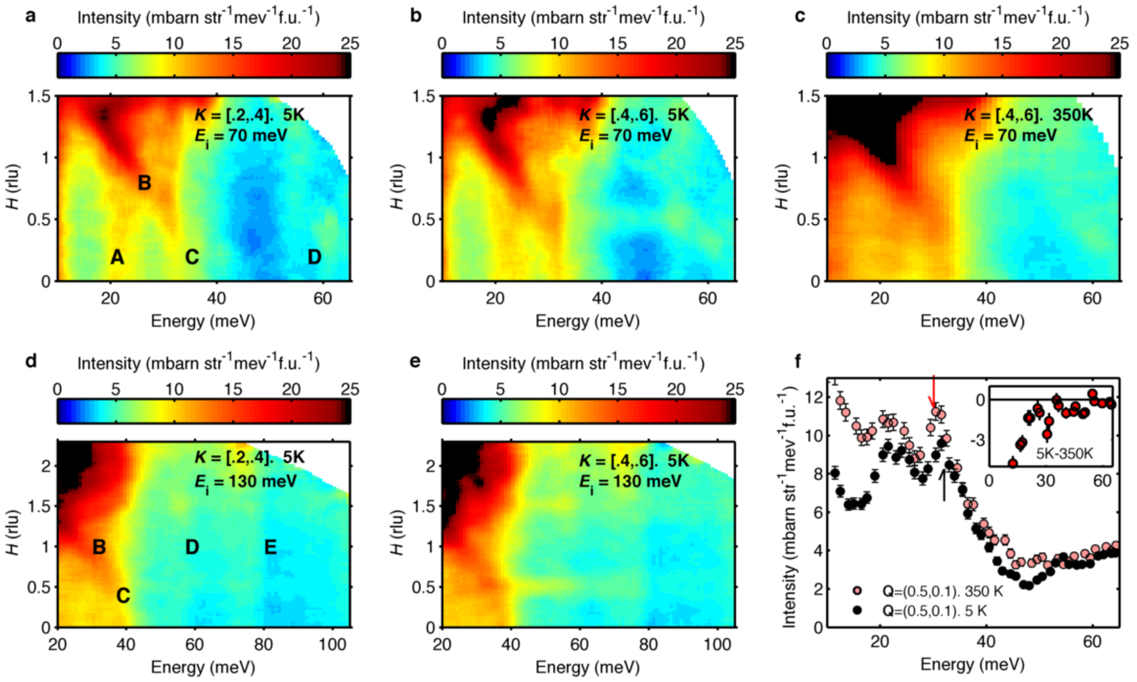


**Supplementary Figure 3 | Additional contributions to the measured intensity. (a-b)** Energy dependence of total scattering intensity at $T$ = 5 K, with $E_i$ = 70 meV, along the momentum transfer trajectories [$H$, 0.3±0.1] and [$H$, 0.5±0.1], respectively. The letters A-D in **a** mark various contributions to the scattering besides the AF fluctuations that we are concerned with: A and C are optical phonons with nearly zero dispersion; B is a dispersing phonon from a powder contribution, probably Aluminum, and presents as a ring of scattering in constant-energy slices, as demonstrated in Supplementary Fig. 5; D is one of the weakly-dispersing modes reported previously for the pseudogap phase of Hg1201 (Supplementary refs. 5 and 6). In **b**, AF fluctuations centered at $\mathbf{q}_{AF}$ = (0.5, 0.5) are clearly seen in the raw data superimposed on the "background" contributions A-D. As demonstrated in Fig. 2 of the main text, the AF fluctuations can be successfully isolated by subtracting the various additional contributions. However the scattering within the energy range 30-36 meV around $\mathbf{Q}_{AF}$ is typically highly contaminated by the Aluminum

and phonon lines, which results in a slight overestimation of the magnetic contribution. (**c**) Same as **b**, but at 350 K, above the pseudogap temperature. The contributions A-C are more intense, whereas D is no longer observed. This confirms the identification of A-C as phonons and D as a possible magnetic mode[5-6]. (**d-e**) Same as **a&b**, but with $E_i$ = 130 meV. The features B-D can be identified. An additional dispersionless feature is observed at ~ 75 meV, marked by E. Although high-temperature data were not taken with $E_i$ = 130 meV, unpublished measurements at 420 K on a more underdoped Hg1201 sample ($T_c \approx 55$ K, $T^* =$ 400 K) show that, unlike the mode at 55 meV (feature D) this dispersionless feature is still observed at high temperatures. The origin of this feature is undetermined. (**f**) Energy dependence of scattering at **q** = (0.5, 0.1) for 5 K and 350 K, with $E_i$ = 70 meV. The 30 meV phonon (marked by the red and black dashed lines) softens by about 1-2 meV with increasing temperature.

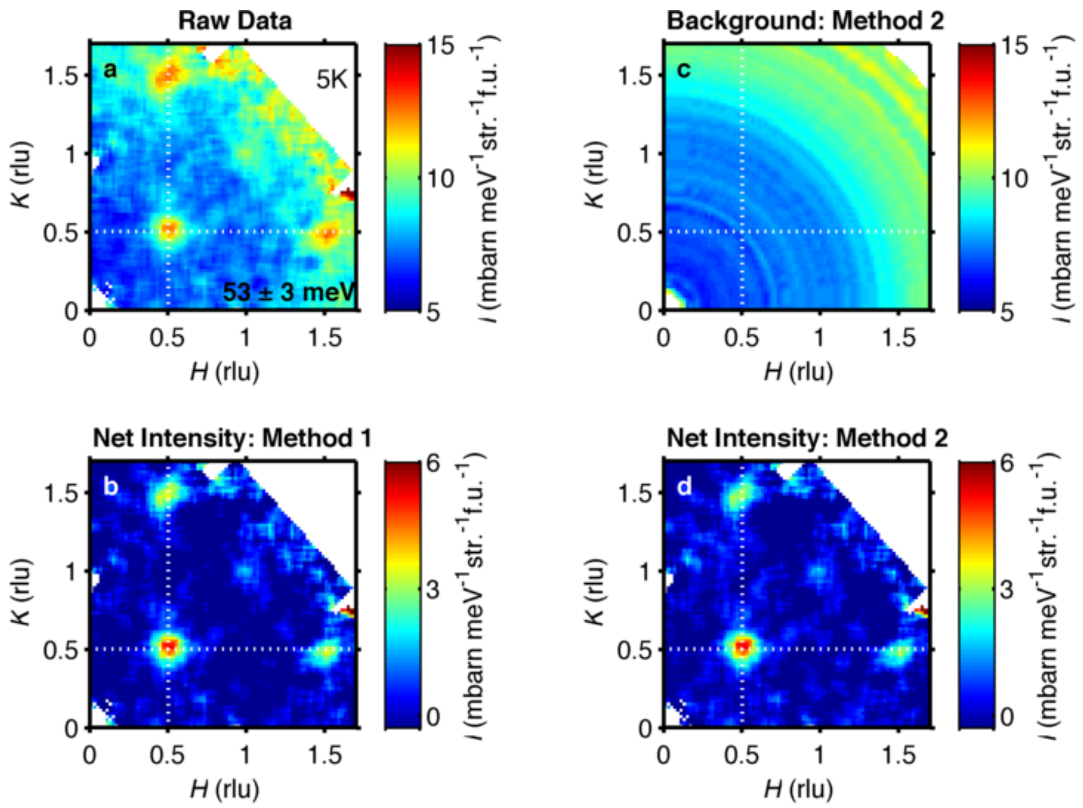


**Supplementary Figure 4 | Data processing – Part I.** (**a**) Representative constant-energy slice ($\omega$ = 53 ± 3 meV) of the raw data. In order to isolate the AF fluctuations, two possible methods are employed. (**b**) Result of implementing Method 1. (**c**) Calculated "background" level determined from Method 2. (**d**) Resultant net intensity with Method 2. For this particular energy slice, both methods produce essentially equivalent results. As demonstrated in Supplementary Fig. 5, Method 2 must be used when spurious contributions from powder lines are present.

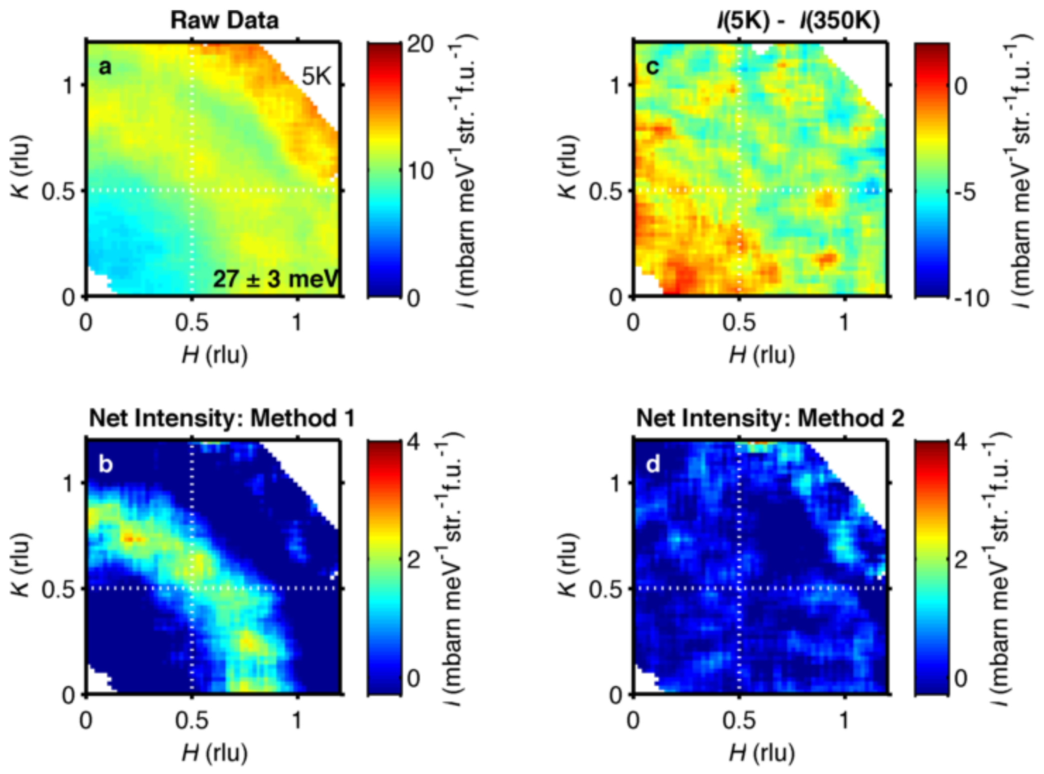


**Supplementary Figure 5 | Data processing – Part II.** (**a**) Example of a constant-energy slice ($\omega$ = 27 ± 3 meV) in which a powder ring obscures the underlying behavior at $\mathbf{q}_{AF}$. (**b**) Net intensity after subtracting the "background" contributions determined using Method 1. It is clear that this method does not adequately remove the spurious powder ring. (**c**) The non-AF contributions visible in the raw data can be largely removed by taking the temperature difference between data at 5K and 350 K. (**d**) Method 2 successfully removes the powder ring without taking a temperature difference. Consistent with the discussion in the main text, no AF fluctuations are observed for $\omega \approx 27$ meV.

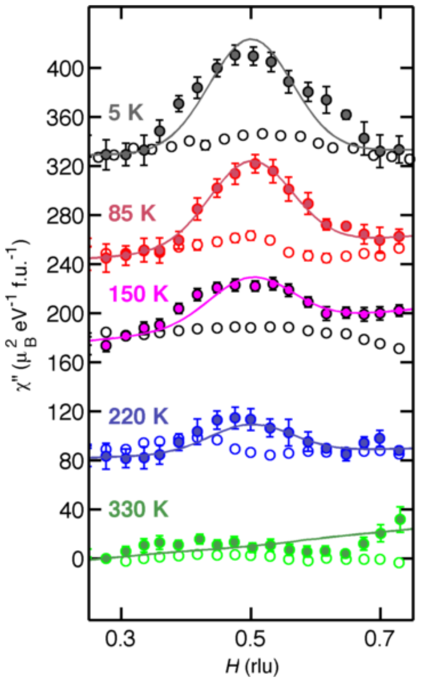


**Supplementary Figure 6 | Temperature dependence of the low-energy response.** Temperature dependence of the magnetic susceptibility at $\omega$ = 36 ± 3 meV (solid symbols) and 15 ± 5 meV (open symbols). Cuts along along {100} and {010} are averaged. Solid lines are Gaussian fits to the $\omega$ = 36 meV data.

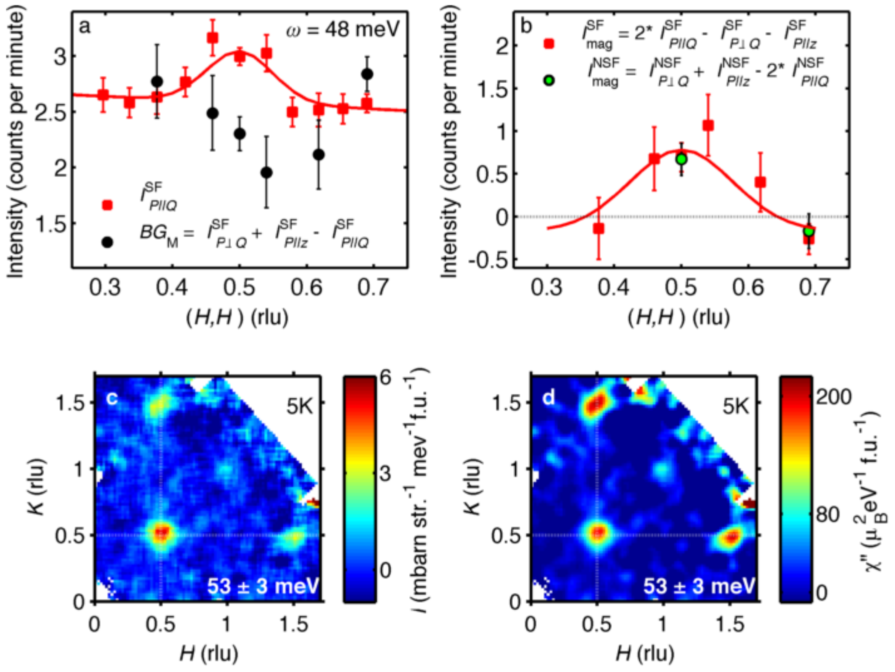


**Supplementary Figure 7 | Verification of magnetic scattering.** (**a**) Rocking-scan of spin-flip (SF) scattering about **Q** = (0.5, 0.5, 3.5) at *ω* = 48 meV and *T* = 2 K (red squares) for initial neutron spin polarization **P** parallel to **Q** ($I^{\mathrm{SF}}_{P\|Q}$ ). SF scattering measures magnetic fluctuations perpendicular to both **Q** and **P**, as well as incoherent nuclear spin scattering and background contributions. The background level for magnetic scattering defined as $BG_M \equiv \left(\frac{2}{3}N_{\mathrm{inc,spin}} + BG\right)$ is determined in longitudinal polarization analysis (black circles) through additional measurements in the two other principal geometries ($I^{\mathrm{SF}}_{P\|z}$ and $I^{\mathrm{SF}}_{P\perp Q}$). Excess scattering above the background level, which we attribute to magnetic fluctuations, is clearly observed at $\mathbf{q}_{\mathrm{AF}}$. (**b**) The intensity measured in the three SF geometries can be used to extract the pure magnetic scattering (red squares). This is confirmed through a corresponding NSF measurement (green circles). Error bars in **a** and **b** represent statistical uncertainty (1 s.d.). (**c**) Constant-energy (*ω* = 53 ± 3 meV) slice from TOF measurement showing magnetic scattering intensity in several

Brillouin zones. The distinct peak at **Q** = (0.5, 0.5, 4.55) is repeated at **Q** = (1.5, 0.5, 6.34) and (0.5, 1.5, 6.34). (**d**) The data from **c** are normalized by the anisotropic magnetic form factor for Cu $3d_{x^2-y^2}$ (ref. 2) to obtain $\chi''$.

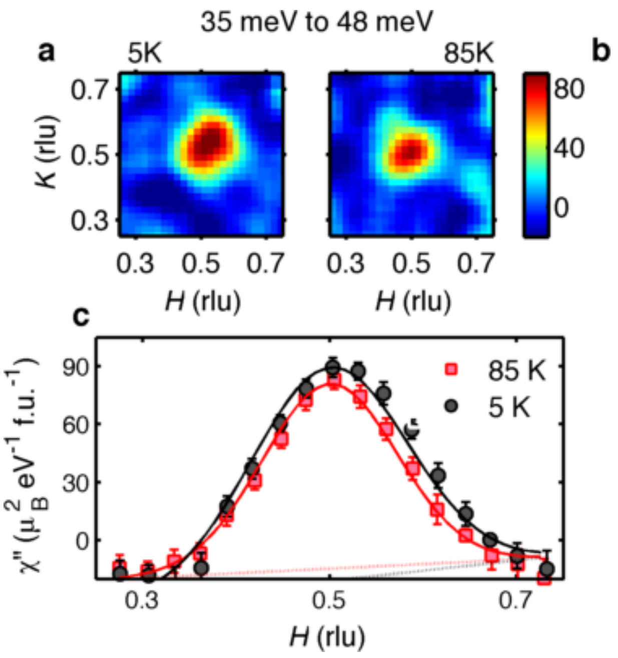


**Supplementary Figure 8 | Effect of superconductivity on the width of the low-energy response.** (**a**),(**b**) $\chi''(\mathbf{Q})$ constant energy slice integrated between 35 meV and 48 meV at 5 K and 85 K respectively. (**c**) Corresponding cut of the data averaged over the {100} and {110} trajectories of the data in (a) and (b). The peak at 5 K is slightly broader than that at 85 K, which accounts for the enhancement at $\omega_1$ away from $\mathbf{q}_{AF}$ in $\Delta\chi'' = \chi''(5\text{ K}) - \chi''(85\text{ K})$ discussed in Fig. 3d.

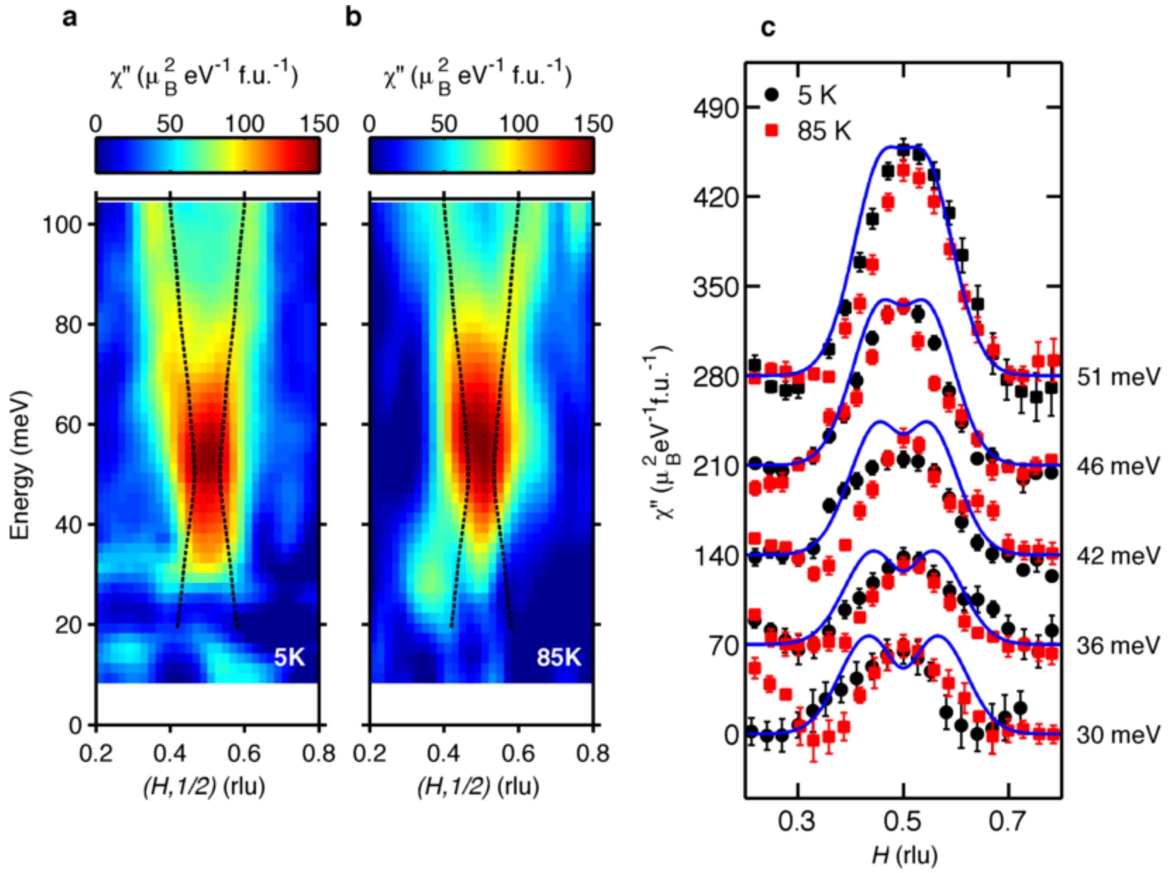


**Supplementary Figure 9 | Comparison to LSCO** (**a-b**) Energy dependence of $\chi''(\mathbf{Q},\omega)$ for HgUD71 at 5 K and 85 K along the two-dimensional momentum-transfer trajectory [$H$, 0.5] with $E_i$ = 130 meV, reproduced from Fig. 2 of the main text. Dotted black lines show the dispersion of LSCO ($p$ = 0.085)[8] shifted to higher energies by 17 meV such that the neck of the hourglass coincides with the peak in magnetic scattering at 51 meV for HgUD71, as expected based on the phenomenology of the cuprates[9,10]. Whereas the high-energy ($\omega$ > 50 meV) dispersion for HgUD71 is similar to that for LSCO, this is not the case for the low-energy response, which exhibits an outward dispersion for LSCO and is commensurate for HgUD71. (**c**) Constant-energy cuts with {100} and {010} trajectories averaged to improve statistics. Due to the better counting statistics, data obtained with $E_i$ = 70 meV rather than $E_i$ = 130 meV are shown. The data are offset vertically for clarity.

The blue lines show the hypothetical response for LSCO for the hourglass dispersion away from the neck indicated in (a) and (b) with FWHM = 0.12 r.l.u. (consistent with our fit result) and convolved with the experimental resolution for $E_\mathrm{i}$ = 70meV . The FWHM was chosen to approximate that determined for HgUD71 in the relevant energy range (inset to Fig. 3b in the main text). It is clear that the low-energy response of HgUD71 is not consistent with the dispersion observed for LSCO.

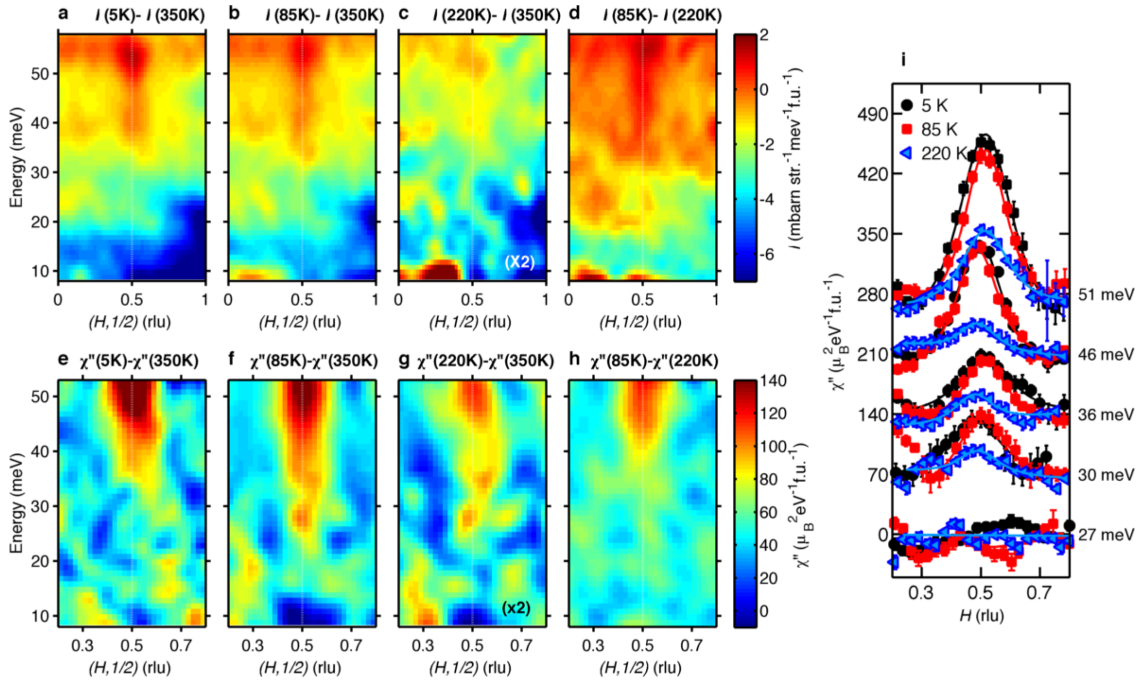


**Supplementary Figure 10 | Temperature dependence of the AF response.** (**a-c**) Raw scattering intensity at $T$ = 5 K, 85 K and 220 K, respectively, after subtracting the data taken at 350 K (shown in Supplementary Fig. 3c). Note that 350 K is above $T$*. The data were taken with $E_\mathrm{i}$ = 70 meV. The intensity in **c** is multiplied by a factor of two to highlight weak features. Both the AF fluctuations and dispersionless modes at about 40 meV and 55 meV are visible. The two Ising-like modes were previously reported for optimally-doped ($T_\mathrm{c}$ ≈ 95 K) and under-doped ($T_\mathrm{c}$ ≈ 65 K) Hg1201[5-6]. (**e-g**) $\chi''(\mathbf{Q},\omega)$ determined by processing the data in **a-c** with Method 2, and converting to the susceptibility. The dispersionless features are absent because they are explicitly removed by this procedure, which isolates the AF response. (**d,h**) Same as above, but with focus on the change of the response between 220 K (just above the CDW temperature) and 85 K (just above $T_\mathrm{c}$). (**i**) Line cuts of the data across $\mathbf{q}_\mathrm{AF}$ after background subtraction as in panels **e-g**. Cuts along {100} and {010} trajectories were averaged to improve statistics. Data at each energy are offset vertically for clarity.

**Supplementary Note 1**

**Analysis of time-of-flight neutron scattering data.** Supplementary Figs. 1 and 2 provide sample and instrument resolution information, respectively. The energy and momentum dependence of the raw measured scattering intensities are shown in Supplementary Fig. 3. Besides the AF fluctuations, manifest as rods of scattering centered at $\mathbf{q}_{AF}$, a number of additional features are observed. These are identified in Supplementary Figs. 3a and 3d. In order to properly analyze the AF fluctuations they have to be isolated from these other contributions. Supplementary Figs. 4 and 5 demonstrate the use of two methods to subtract an energy ($\omega$) and wave-vector ($\mathbf{Q}$) dependent "background" $BG(\omega,\mathbf{Q})$. Method 1 fits a constant-energy slice to a second-order polynomial in the magnitude of the wave vector: $BG(\omega,\mathbf{Q}) = A(\omega) + B(\omega)|\mathbf{Q}| + C(\omega)\mathbf{Q}^2$. In Method 2, $BG(\omega,\boldsymbol{Q})$ is taken to be the average of the intensity at a given wave-vector magnitude $|\mathbf{Q}|$ at fixed energy transfer. In both methods, the data around $\mathbf{q}_{AF}$ are excluded from the determination of the additional contributions. Although Method 1 is more straightforward to implement, Method 2 is required to remove scattering from powder lines. This is evident from the comparisons in Supplementary Figs. 4 and 5. The processed data presented in the main text are based on Method 2 and, where feasible, the results are confirmed with Method 1.

**Supplementary Note 2**

**Obtaining absolute units of the dynamic magnetic susceptibility**. TOF data are converted to absolute units by comparison with a Vanadium standard. Assuming isotropic magnetic fluctuations, the AF scattering cross-section is related to the imaginary part of

the dynamic magnetic susceptibility, $\chi''(\mathbf{Q},\omega)$, via[2]

$$\frac{d^2\sigma}{d\Omega dE} = \frac{2(\gamma r_e)^2}{\pi g^2 \mu_B^2}\frac{k_\mathrm{f}}{k_\mathrm{i}}|F(\mathbf{Q})|^2\frac{\chi''(\mathbf{Q},\omega)}{1-\exp(-\omega/k_B T)}$$

where $(g r_\mathrm{e})^2 = 0.2905$ barn $\mathrm{sr}^{-1}$, $k_\mathrm{f}$ and $k_\mathrm{i}$ are the final and incident neutron wave-vectors, and $|F(\mathbf{Q})|^2$ is the magnetic form factor. Supplementary Fig. 7c shows a constant energy image with peaks of intensity at $\mathbf{q}_\mathrm{AF}$ at $\omega = 53$ meV. The scattering intensity is larger at $\mathbf{Q} = (0.5, 0.5, 4.55)$ than in higher (two-dimensional) Brillouin zones ($\mathbf{Q} = (0.5, 1.5, 6.34)$ and (1.5, 0.5, 6.34)), consistent with magnetic scattering, for which the form-factor is smaller at larger $\mathbf{Q}$. After accounting for the anisotropic $Cu^{2+}$ $d_{x^2-y^2}$ form factor[2], $\chi''$ becomes equivalent in all zones (Supplementary Fig. 7d).

**Supplementary Note 3**

**Limits on the low-energy response**. Supplementary Fig. 6 shows the temperature dependence of response at $\omega = 15 \pm 5$ meV and $\omega = 36 \pm 3$ meV. There is no discernible magnetic scattering above the concave background at $\omega = 15$ meV, and we estimate conservative upper bounds of 15% and 8%, respectively, compared to the signal at $\omega = 36$ meV and 51 meV. We note that $\chi''_0(\omega)$ for $\omega > \Delta_\mathrm{AF}$ extrapolates to zero at $\omega \sim 25$ meV (Fig. 3a; 5 K data), consistent with $\Delta_\mathrm{AF} = 27$ meV, which is defined as the energy below which we can no longer observe a peak at $\mathbf{Q}_\mathrm{AF}$.

**Supplementary Note 4**

**Polarized neutron scattering.** Spin-polarized measurements (Supplementary Fig. 7a,b) were carried out on the triple-axis spectrometer IN20 at the Institute Laue Langevin.

Heusler alloy crystals were used as monochromator and analyzer to select the initial and final neutron energies and spin polarizations. The polarization of the neutron beam in the vicinity of the sample was maintained by CryoPAD, which provides high stability and reproducibility of the neutron spin polarization[4].

For longitudinal polarization analysis, it is convenient to define the coordinate system based on the relative orientations of the neutron spin polarization (**P**) at the sample, the scattering wave-vector **Q**, and the scattering plane that contains **Q**: the three orthogonal axes are defined by **P**||**Q** and **P** ⊥ **Q** in the scattering plane, and **P**||**z**, where **z** is the direction perpendicular to the scattering plane. In the absence of chiral magnetic correlations, the measured spin-flip (SF) and non-spin-flip (NSF) scattering intensities in the three principal spin-polarization geometries are given by the following relations[1]:

$$I_{P\parallel Q}^{\mathrm{SF}} = \frac{2}{3}N_{\mathrm{inc,spin}} + M_{P\perp Q} + M_{P\parallel z} + BG \quad (1)$$

$$I_{P\parallel Q}^{\mathrm{NSF}} = N_{\mathrm{coh}} + N_{\mathrm{inc,isotope}} + \frac{1}{3}N_{\mathrm{inc,spin}} + BG \quad (2)$$

$$I_{P\perp Q}^{\mathrm{SF}} = \frac{2}{3}N_{\mathrm{inc,spin}} + M_{P\parallel z} + BG \quad (3)$$

$$I_{P\perp Q}^{\mathrm{NSF}} = N_{\mathrm{coh}} + N_{\mathrm{inc,isotope}} + \frac{1}{3}N_{\mathrm{inc,spin}} + M_{P\perp Q} + BG \quad (4)$$

$$I_{P\parallel z}^{\mathrm{SF}} = \frac{2}{3}N_{\mathrm{inc,spin}} + M_{P\perp Q} + BG \quad (5)$$

$$I_{P\parallel z}^{\mathrm{NSF}} = N_{\mathrm{coh}} + N_{\mathrm{inc,isotope}} + \frac{1}{3}N_{\mathrm{inc,spin}} + M_{P\parallel z} + BG \quad (6)$$

where $N_{\text{inc,isotope}}$ and $N_{\text{inc,spin}}$ are the nuclear isotope and spin incoherent cross sections, respectively, $N_{\text{coh}}$ is the coherent nuclear cross section, $M$ is the magnetic cross section, and $BG$ is the background contribution. Supplementary Fig. 7a shows excess scattering in $I_{P\parallel Q}^{\text{SF}}$ above the background level at the two-dimensional AF wave vector at $\omega$ = 48 meV. We attribute this to scattering from magnetic fluctuations. To further confirm this, we measure all six SF and NSF neutron polarization configurations for select wave vectors and extract the pure magnetic intensity from both SF and NSF channels: $M_{P\perp Q}+M_{P\parallel z}=2\times I_{P\parallel Q}^{\text{SF}}-I_{P\perp Q}^{\text{SF}}-I_{P\parallel z}^{\text{SF}}=I_{P\perp Q}^{\text{NSF}}+I_{P\parallel z}^{\text{NSF}}-2\times I_{P\parallel Q}^{\text{NSF}}$. Supplementary Fig. 7b shows unambiguous evidence of magnetic scattering at $\mathbf{q}_{\text{AF}}$ from both SF and NSF scattering.

**Supplementary Note 5**

**Effect of superconductivity on the magnetic susceptibility**. HgUD71 does not exhibit a SC resonance. However, we observe subtle changes of the susceptibility ($\Delta\chi''$) in the SC phase at wave vectors away from $\mathbf{q}_{\text{AF}}$ (Figs. 3b,d and Supplementary Fig. 8). $\Delta\chi''$ around $\omega_1$ is due to a slight increase of momentum width at 5 K (Figs. 1b,c and Fig. 3b inset and Supplementary Fig. 8), whereas at $\omega_2$ it results from an increase in amplitude on the upward dispersive part of the spectrum (Figs. 2c,h,m).

**Supplementary Note 6**

**Temperature dependence of the commensurate response**. Supplementary Fig. 10 shows the evolution of the low-energy magnetic response across the temperatures $T_c$ = 71 K and $T_{\text{CDW}}$ = 200 K (ref. 7) for HgUD71. In order to reduce background contributions,

the scattering at 350 K is first subtracted from that at 5 K, 85 K and 220 K (Supplementary Figs. 10a,b,c). The gapped commensurate $\mathbf{q}_{AF}$ response is already observed in the (H,1/2) vs. energy slices. The background subtraction procedure described in Supplementary Note 1 is then applied at all energies to more clearly isolate the AF fluctuations in Supplementary Figs. 10e,f,h. Besides the slightly broader response at 5 K described in the main text, the overall commensurate spectrum remains largely impervious to the onset of superconductivity and CDW order. This is also apparent from the line cuts of the data in Supplementary Figs. 10i. Supplementary Figs. 10d,h shows the enhancement of the response between $T_{CDW}$ and $T_c$. The lack of significant changes in the **Q**-ω dependence or in the scattering intensity (Fig. 1a) across $T_{CDW}$ and $T_c$ highlights that the commensurate low-energy spectrum is a signature of the PG state which has higher characteristic temperature $T^*$ ($T_c < T_{CDW} < T^*$).

## Supplementary References


1. Abernathy, D. L. *et al.* Design and operation of the wide angular-range chopper spectrometer ARCS at the Spallation Neutron Source. *Rev. Sci. Instrum.* **83**, 015114 (2012).
2. Lovesey, S. W. *Theory of neutron scattering from condensed matter*, vol. 2 (Oxford, 1984).
3. Shamoto, S., Sato, M., Tranquada, J. M., Sternlieb, B. J., & Shirane, G. Neutron-scattering study of antiferromagnetism in $YBa_2Cu_3O_{6.15}$. *Phys. Rev. B* **48**, 13817 (1993).
4. Tasset, F. *et al.* Spherical neutron polarimetry with Cryopad-II. *Physica B* **267**, 69-74 (1999).
5. Li, Y. *et al.* Hidden magnetic excitation in the pseudogap phase of a model cuprate superconductor. *Nature* **468**, 283-285 (2010).
6. Li, Y. *et al.* Two Ising like collective magnetic excitations in a single-layer cuprate superconductor. *Nat. Phys*. **8**, 404-410 (2012).
7. Tabis, W. *et al.* Connection between charge-density-wave order and charge transport in the cuprate superconductors. *Nat. Commun.* **5**, 5875 (2014).
8. Lipscombe, O. J., Vignolle, B., Perring, T. G., Frost, C. D., & Hayden, S. M. Emergence of coherent magnetic excitations in the high temperature underdoped superconductor $La_{2-x}Sr_xCuO_4$ at low temperatures. *Phys. Rev. Lett.* **102**, 167002 (2009).
9. Hayden, S. M. *et al*. The structure of the high-energy spin excitations in a high-transition temperature superconductor. *Nature* **429**, 531-534 (2004).
10. Tranquada, J. M. *et al.* Quantum magnetic excitations from stripes in copper oxide superconductors. *Nature* **429**, 534-538 (2004).
